# A Case Study on Neural Activity Characteristics in a Shooting Competition

**DOI:** 10.3390/brainsci15020174

**Published:** 2025-02-10

**Authors:** Zijin Li, Meiliang Liu, Zhengye Si, Junhao Huang, Yunfang Xu, Zhiwen Zhao

**Affiliations:** 1 School of Artificial Intelligence, Beijing Normal University, Beijing 100091, China; 202131081021@mail.bnu.edu.cn (Z.L.); 202231081016@mail.bnu.edu.cn (M.L.); 202231081017@mail.bnu.edu.cn (Z.S.); z1x213178897308@163.com (J.H.); 202031210018@mail.bnu.edu.cn (Y.X.); 2 Advanced Institute of Natural Sciences, Beijing Normal University, Zhuhai 519087, China

**Keywords:** shooting competition, EEG, neural activity, PSD, PLV

## Abstract

**Background**: Sexual characteristics in brain neurophysiological activity are a significant area of research in cognitive neuroscience. As a sport that involves minimal physical movement, shooters remain largely stationary during aiming, facilitating the collection of their neural activity compared to athletes in other sports. **Objectives**: To investigate the neural characteristics of novice shooters of different genders under competitive conditions. **Methods**: Sixteen subjects participated in a shooting competition following four weeks of training. Electroencephalogram (EEG) data and behavioral data (shooting scores, aiming curves, and pressure curves) were recorded during the competition, and the power spectral density (PSD) and phase-locking value (PLV) network features were extracted to explore further the correlation between the shooting scores and neural activity. **Results**: In our sample, (1) there were no significant differences in shooting scores between males and females; (2) there were differences in PSD values across the theta, alpha, alpha-2, beta, and gamma frequency bands between males and females; and (3) there were differences in PLV network properties in the theta, alpha, beta, and gamma frequency bands between males and females. Correlation analysis revealed associations between shooting scores and neural activity in male and female novices. **Conclusions**: The case study demonstrated that males and females exhibited different neural activity characteristics in the shooting competition, providing a foundation for further investigation into the sex differences in neural activity in shooting competition.

## 1. Introduction

Sexual characteristics in neural activity have been an essential area of research in neuroscience. Comparing neural activity between age-matched male and female groups may help elucidate the neural mechanisms underlying sex differences [1,2,3,4]. Attention is a key focus in cognitive neuroscience. As a closed sport [5], shooting requires relatively little physical effort, with athletes remaining largely stationary during aiming, as demonstrated by López et al. [6], which makes it an ideal task for investigating neural activity during intense concentration compared to other sports [7,8,9]. Current research on shooting focuses on two areas: the neural mechanisms involved in aiming by professional shooters and the differences in neural activity between experienced and novice shooters [10].

Many studies have investigated the relationship between shooting skills and neural activity parameters [11,12,13,14,15,16]. During the aiming period of a shooting task, individual behavior is closely associated with distinct brain electroencephalogram (EEG) rhythms. Hatfield et al. reported a significant increase in alpha activity in the left temporal and occipital regions of athletes during the aiming period [11]. Loze et al. observed the alpha power characteristics of expert shooters before shooting [12]. Doppelmayr et al. investigated the theta activity in the frontal midline of experts and novices during aiming [14]. Gong et al. measured the resting EEG activity of rifle athletes, and they found that the characteristic path length (CPL) showed a significant correlation [10]. Badarin et al. analyzed the correlation between the EEG data of male novices and shooting scores [17]. These studies have indicated significant correlations between shooting scores and neural activity characteristics. Meanwhile, Legault et al. investigated the sex differences in perceptual-cognitive learning among young athletes, finding that male athletes demonstrated superior perceptual abilities compared to female athletes, and the benefits of sport-related activities on brain function, with an even more significant impact on females [18]. Vona et al. assessed the cognitive task performance of elite athletes of different sexes, finding that males performed significantly faster than females on motor reaction time measures of attention and inhibition [19]. Previous studies [10,11,12,13,14,15,16,17] have examined the neural activity features of athletes during aiming and analyzed the correlation between shooting scores and neural activity. Furthermore, other studies [18,19] have compared the sexual differences between athletes. However, there has hardly been any investigation of neural activity characteristics in male and female novices during the rifle aiming period. Therefore, our study aimed to investigate the neural activity features of novice shooters during a shooting competition, providing a foundation for future research to explore the sexual differences in novices in a similar scenario.

## 2. Materials and Methods

### 2.1. Subjects

We conducted a priori power analysis by G* Power [20], with a power of 0.8, an alpha level of 0.05, and an effect size of 0.25 [21]. This analysis estimated that 28 volunteers were needed for this competition. However, due to the longer training period and strict training requirements, we only managed to recruit 20 volunteers. During the training, four of the participants withdrew due to training pressure. Moreover, we conducted a prior survey of the female subjects and scheduled the competition to avoid coinciding with their menstrual cycles. A college shooting competition, sponsored by the Zhuhai Computer Federation, involved sixteen college students (6 males and 10 females) from Beijing Normal University Zhuhai Campus, averaging 21.2 (±0.2) years. No participants had prior shooting experience. Before the competition, participants underwent 4 weeks of training. All were right-handed, with normal or corrected-to-normal vision and normal hearing. They maintained regular sleep schedules and exhibited good mental health. They also refrained from consuming stimulants (e.g., alcohol, coffee, tea, or any neurological drugs) for 24 h before the competition to avoid potential influence on the results. This study was conducted following the guidelines of the Declaration of Helsinki and approved by the Ethics Committee of Beijing Normal University (IRB number: BNU202405150103). All participants voluntarily joined the competition, understood its purpose and procedures, and signed informed consent forms beforehand. They were also informed that they could report any discomfort and withdraw from the competition at any time. Inclusion criteria were as follows: age requirement: participants were between 18 and 22 years old; sex requirement: both male and female were accepted; health status: participants had no history of neurological or psychological disorders and no severe visual or hearing impairments; uncorrected or corrected visual acuity of 1.0 or above; shooting experience: participants were novice shooters (i.e., had no prior shooting experience); right-handedness: all participants were right-handed; voluntary participation: participants voluntarily took part in the study and signed an informed consent form. The exclusion criteria were as follows: significant loss of vision or hearing; neurological or mental illness; medication that affects the nervous system taken 3 days before the shooting competition; individuals with prior shooting or archery experience.

### 2.2. Competition Environment Setting

**Figure 1:**
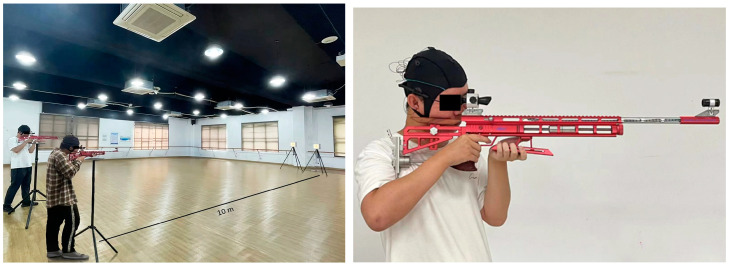
The shooters aimed and shot at a target 10 m away while the EEG data were recorded.

The competition was conducted indoors with comfortable lighting. Each participant used a laser rifle to shoot at a target 10 m away. The material and weight of the rifle used in our experiment are identical to those used in the air rifles employed in the Olympics. The laser rifle used in our competition is made from high-quality aluminum alloy and carbon fiber materials. The maximum weight of the rifle shall not exceed 5.5 kg, and the caliber of the rifle is 0.177 inches. The only difference is that our equipment employs invisible infrared lasers instead of bullets. Moreover, all shooters stood and pulled the trigger with their index finger, as shown in Figure 1.

### 2.3. Procedure

The top two shooters with the highest total scores in the competition were awarded substantial rewards to incentivize participation. The competition comprised multiple rounds, as outlined below:

1. Initial Round: Each shooter took 15 shots within 450 s, with each shot requiring aiming and trigger pulling within 30 s. Following this round, the four lowest-ranked shooters were excluded.
2. Second Round: The remaining twelve shooters took 5 shots within 150 s. The four lowest-ranked shooters were disqualified.
3. Third Round: The eight remaining shooters took 5 shots. The two lowest-ranked shooters were excluded.
4. Fourth Round: The six remaining shooters took 5 shots. The two lowest-ranked shooters were eliminated.
5. Fifth Round: The four remaining shooters took 5 shots within 150 s. The two lowest-ranked shooters were disqualified, leaving the final two shooters to compete for the championship.
6. Final Round: The two remaining shooters took 5 shots within 150 s, and the winner was determined by their total scores.

**Figure 2:**
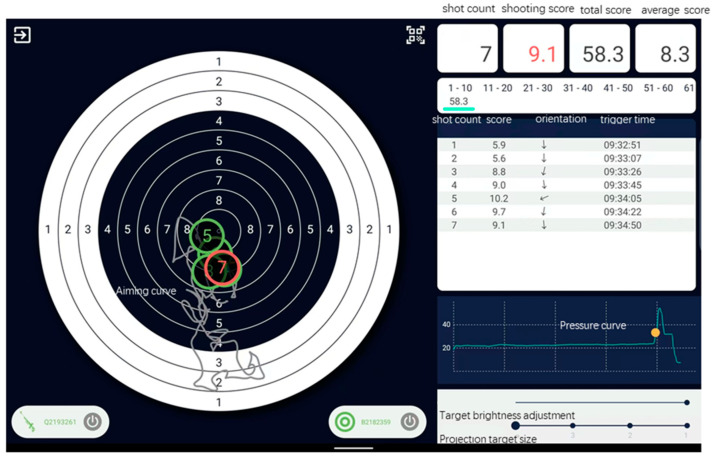
The shooting system recorded each shot’s score, aiming, and pressure curve.

Figure 2 shows that shooting scores, aiming curves, and pressure curve data were recorded during the competition.

Additionally, we recorded EEG data during the competition, marking EEG data according to trigger pulling. We synchronized the EEG data with the trigger-pulling events based on the records.

### 2.4. Methods

EEG signals were collected at rest, and during aiming, the power spectral density (PSD) and phase-locking value (PLV) network topology were analyzed. The correlations between these neural activity characteristics and shooting scores were also examined. By analyzing EEG signals during aiming, we hypothesized that there are differences in the neural activity characteristics of novice shooters based on sex and that these neural activity characteristics would affect novice shooters’ scores.

#### 2.4.1. EEG and Behavioral Data Acquisition

The EEG acquisition device, Unicorn Hybrid Black (g.tec Medical Engineering GmbH, Schiedlberg, Austria), is a portable wireless EEG system that connects to a computer via Bluetooth. Given the potential signal reliability concerns of dry electrodes, we applied the conductive paste to enhance electrode conductivity to improve signal quality during the competition. The device has a sampling rate of 250 Hz. The device has a resolution of 24 bits, with an impedance greater than 100 MΩ. Following the 10–20 international standard, the electrode positions of the device are Fz, C3, Cz, C4, Pz, PO7, Oz, and PO8. These electrodes cover key brain areas, including the frontal, parietal, temporal, and occipital lobes. Unicorn Suite hybrid black (1.18.00) software was employed to establish a successful Bluetooth connection between the Unicorn and the PC and to provide real-time data visualization, storage, and playback functions [22]. Before the competition began, the electrode impedance was adjusted and maintained low enough to ensure smooth EEG signals. EEG data from the entire competition process were collected and recorded.

The competition used a laser rifle training system manufactured by Zhuhai Qiangyuan Technology Co., Ltd., Zhuhai, China. The shooting score, aiming jitter curve, and pressure curve for each shot were displayed on the screen in real-time and saved in the database.

#### 2.4.2. EEG Data Preprocessing

The EEG signals were preprocessed in MATLAB R2020b using EEGLAB v2021.0. First, a finite impulse response (FIR) filter was applied for bandpass filtering with a range from 0.1 to 100 Hz, and a 50 Hz notch filter was set to remove power-line frequency noise [23]. The EEG data were re-referenced, and the first 2 s of the epochs corresponding to the aiming period were extracted based on the marks. After reviewing the EEG data, flawed trials were excluded. Noise and artifacts in the raw EEG data, which represent unwanted information, were removed using the independent component analysis (ICA) of EEGLAB [24,25]. Eye blinks, eye movements, muscle activity, and power frequency interference cause artifacts in EEG data. The ICLabel tool automatically labeled and removed components associated with channel noise, line noise, eye movement, and muscle artifacts [26]. In this study, we analyzed the theta (5–7 Hz), alpha (8–13 Hz), beta (15–29 Hz), and gamma (30–100 Hz) frequency bands. Brainstorm (Brainstorm_230322) software was used to compute the PSD and PLV of the EEG data.

#### 2.4.3. EEG PSD Analysis

Welch’s method (employing a Hamming Window) [27] was applied to analyze the PSD in dB using Brainstorm. The window length was 2 s, with a 50% overlap. The PSD provides information on relative energy distribution within the EEG signal across different frequency ranges. Gong’s results indicated that EEG data collected 2 s before the trigger pull exhibited the strongest correlation with the shooting score [28]. The 2 s EEG data collected before the trigger pull were used to calculate the PSD. The individual alpha frequency (IAF) of the EEG data for each shot was determined by identifying the highest peak amplitude within the 8–13 Hz alpha range [29,30]. Based on the IAF, the alpha frequency was further subdivided into alpha-1 (8–10 Hz) and alpha-2 (10–13 Hz). Jaquess et al. indicated that the amplitude of alpha-2 is a marker of conscious self-regulation of cognitive and affective functions [31]. Since shooting requires conscious self-control, we also calculated the correlations between alpha-2 PSD values and shooting scores in male and female subjects.

#### 2.4.4. EEG Functional Connectivity Based on the PLV Network

The PLV measures the phase synchrony between brain activity recorded from two electrodes [32]. The PLV value ranges from zero to one, where zero indicates complete randomness of phase angles, and one indicates the perfect synchronization of phase angles at a given frequency and time point. In this study, we employed brain network analysis to examine the topological characteristics of the PLV network within the functional connection matrix of novices during the shooting competition. Based on the functional connectivity matrix, the network properties of each shot were calculated using the Brain Connectivity Toolbox (BCT, Version: 2019-03-03). The EEG network was quantitatively measured using network properties, including the clustering coefficient (Cc), global efficiency (Eglob), local efficiency (Eloc), and characteristic path length (CPL).

#### 2.4.5. Statistical Analysis

Statistical analysis was performed using SPSS version 22.0. We applied the Shapiro–Wilk (S-W) test to assess whether the shooting EEG and behavioral data followed a normal distribution (see Tables S1–S3). For data following a Gaussian distribution, *t*-tests were conducted, and Pearson’s correlation coefficients were calculated. The Mann–Whitney and Spearman correlation analyses were used for non-Gaussian distributions, as determined by the S-W test. Based on the analysis results, *p*-values of 0.05 and 0.01 were considered to indicate significant differences and very significant differences between the two groups, respectively.

## 3. Results

### 3.1. Shooting Score by Sexes

**Figure 3:**
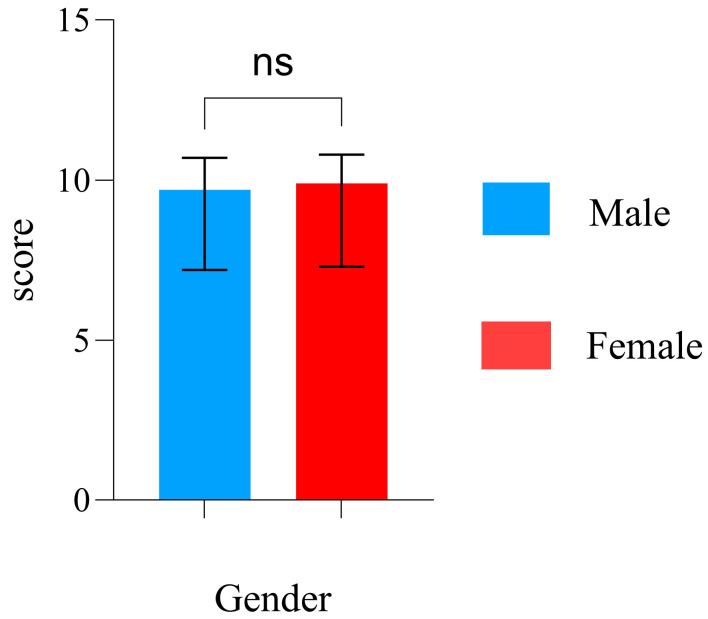
Shooting scores of male and female subjects (ns indicates *p* > 0.05).

Figure 3 shows that the median shooting scores for the male and female groups were 9.7 and 9.9, respectively. The median score of the male group did not differ significantly from that of the female group (*p* = 0.9795), (Table 1).

### 3.2. Sexual Characteristics in the Variance of Aiming Jitter Curves During Aiming

We collected aiming curve data for 2 s before trigger pulls, recording sixty coordinate points per second using the application. The Euclidean distance of each pair of adjacent coordinate points was calculated. To measure the stability of aiming, we calculated the difference in distance between each pair of adjacent points and assessed the variance of differences. As the variance increased, aiming stability decreased accordingly. As the variance decreased, aiming stability improved. We calculated the variance of the aiming jitter curve (VAJC) during the final 2 s, the penultimate second, and the last second of the aiming phase. Sex differences were observed in the VAJC in this study. However, no significant difference in the variance of the pressure curve during trigger pulls was observed between the male and female groups in this study, (Table 2).

**Figure 4:**
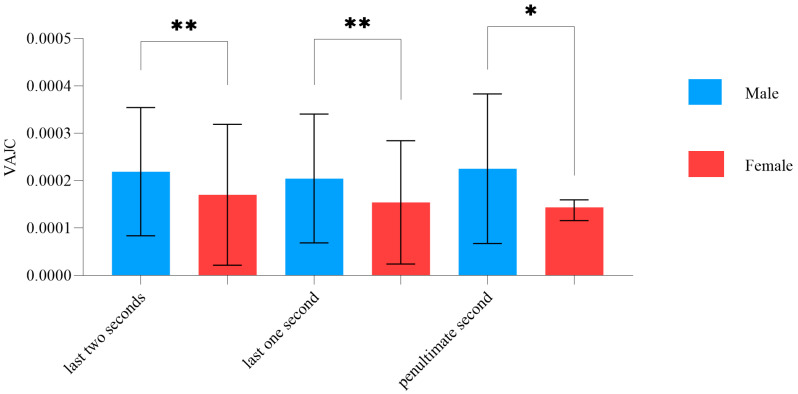
Characteristics in the aiming stability variance (VAJC) between the male and female groups (* indicates *p* ≤ 0.05, ** indicates *p* ≤ 0.01).

Figure 4 illustrates that, in the last two seconds of the aiming curves, the VAJC of males was significantly greater than that for females (*p* < 0.01). In the last and penultimate second of the aiming curves, the VAJC in males was significantly greater than in females (*p* ≤ 0.0132).

### 3.3. Sex Characteristics in PSD

During the aiming phase, the novice shooters’ PSD values were calculated using the Welch method across five frequency bands (theta, alpha, alpha-2, beta, and gamma). The *t*-test and the Mann–Whitney test assessed the differences in PSD values between males and females.

**Figure 5:**
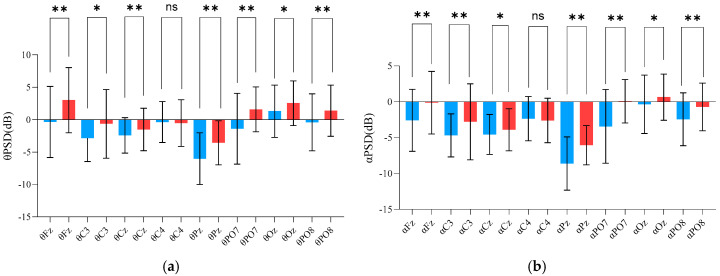

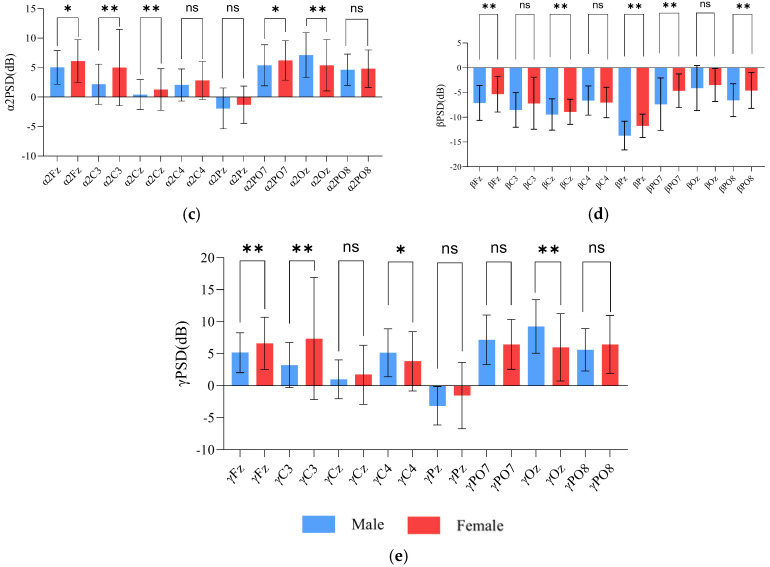
Characteristics in the power spectral density (PSD) (dB) of the five bands (theta, alpha, alpha−2 (10−13 Hz), beta, and gamma) between the male and female groups in the aiming period. (**a**) Different sexual characteristics in the theta PSD, (**b**) different sexual characteristics in the alpha PSD, (**c**) different sexual characteristics in the alpha−2 PSD, (**d**) different sexual characteristics in the beta PSD, and (**e**) different sexual characteristics in the gamma PSD (ns indicates *p* > 0.05, * indicates *p* ≤ 0.05, ** indicates *p* ≤ 0.01).

As shown in Figure 5, in the theta band, the PSD values at Fz, C3, Cz, Pz, PO7, Oz, and PO8 were significantly lower in males than in females (*p* ≤ 0.0149), (Table 3). Similarly, in the alpha band, the PSD values at Fz, C3, Cz, Pz, PO7, Oz, and PO8 were significantly lower in the males (*p* ≤ 0.0433), (Table 4). In the alpha-2 frequency band, the PSD values at Fz, C3, Cz, and PO7 were significantly lower in the males (*p* ≤ 0.0432), while the Oz showed significantly higher values in males (*p* = 0.0049), (Table 5). In the beta band, the PSD values at Fz, Cz, Pz, PO7, and PO8 were significantly lower in the males (*p* ≤ 0.0081), (Table 6). In the gamma band, the PSD values at Fz and C3 were significantly higher in females than males, while the PSD values at C4 and Oz were significantly lower in females compared to the males (*p* < 0.05), (Table 7).

**Figure 6:**
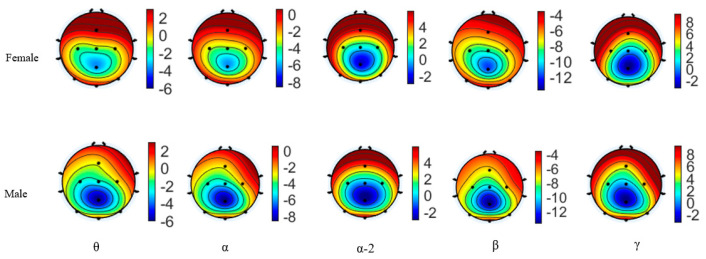
The PSD EEG topographic map of different sexes in theta, alpha, alpha−2, beta, and gamma bands.

We analyzed the brain activation of different sexes in theta, alpha, alpha-2, beta, and gamma bands during aiming, as shown in Figure 6. The top five subfigures are the brain average power of females in theta, alpha, alpha-2, beta, and gamma bands. The bottom five subfigures show the brain average power of males in the same frequency bands. In the theta, alpha, and beta frequency, the female’s PSD values are higher than males. In the alpha-2 frequency band, the PSD values in females are higher than in males, except at the Oz electrode. In the gamma frequency, the PSD values in females are higher than in males, except in the parietal and occipital lobes.

### 3.4. Sex Characteristics in the Functional Connectivity Network During Aiming

During the aiming period, novice shooters’ PLV network values in the theta, alpha, beta, and gamma bands were computed. The *t*-test and the Mann–Whitney test was then used to assess sex characteristics in functional connectivity.

**Figure 7:**
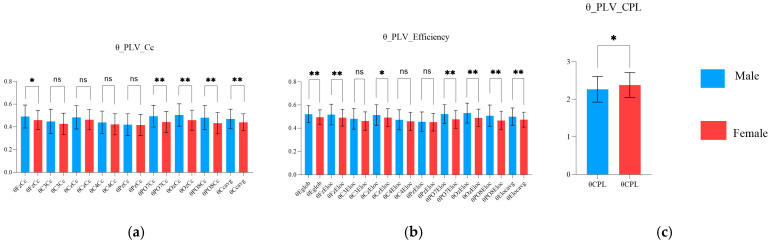
Characteristics in the theta band’s phase-locking value (PLV) network properties between the male and female groups during the aiming period. (**a**) Sex characteristics in theta PLV Cc; (**b**) sex characteristics in theta PLV efficiency; and (**c**) sex characteristics in theta PLV CPL (ns indicates *p* > 0.05, * indicates *p* ≤ 0.05, ** indicates *p* ≤ 0.01).

As shown in Figure 7, in the theta band, the Cc values at Fz, Pz, PO7, Oz, and PO8; the Eglob value; the Eloc values at Fz, Cz, PO7, Oz, and PO8; and the average Cc and Eloc values were significantly higher in the male group than in the female group (*p* ≤ 0.0482). Moreover, the CPL in the theta band was significantly lower in the male group than in the female group (*p* = 0.0152), (Table 8 and Table 9).

**Figure 8:**
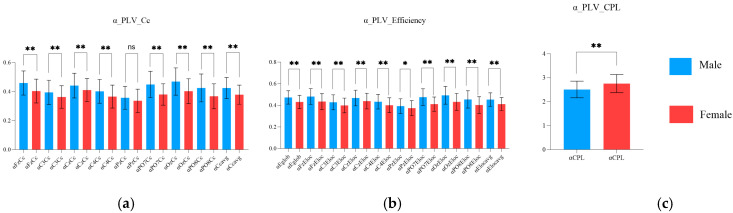
Characteristics of the PLV network properties in the alpha band between the male and female groups during the aiming period. (**a**) sex characteristics in the alpha PLV Cc; (**b**) sex characteristics in the alpha PLV efficiency; and (**c**) sex characteristics in the alpha PLV CPL (ns indicates *p* > 0.05, * indicates *p* ≤ 0.05, ** indicates *p* ≤ 0.01).

As shown in Figure 8, in the alpha band, the Cc values at Fz, C3, Cz, C4, PO7, Oz, and PO8; the Eglob value; the Eloc values at Fz, C3, Cz, C4, Pz, PO7, Oz, and PO8; and the average Cc and Eloc values were higher in the male group than in the female group (*p* ≤ 0.0394). Moreover, the CPL in the theta band was lower in the male group than in the female group (*p* < 0.0001), (Table 10 and Table 11), (Figure 8).

**Figure 9:**
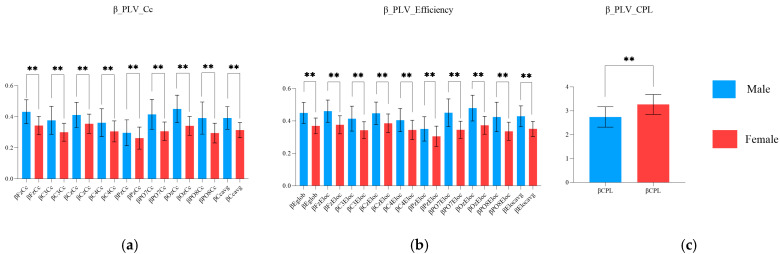
Differences in the properties of the PLV network in the beta band between the male and female groups in the aiming period. (**a**) Sex characteristics in the beta PLV Cc; (**b**) sex characteristics in the beta PLV efficiency; and (**c**) sex characteristics in the beta PLV CPL (** indicates *p* ≤ 0.01).

As shown in Figure 9, In the beta band, the Cc values at all electrodes, the Eglob value, the Eloc values at all electrodes, and the average of Cc and Eloc values were higher in the male group than in the female group (*p* ≤ 0.0025). Moreover, the CPL in the beta band was lower in the male group than in the female group (*p* ≤ 0.0001), (Table 12 and Table 13).

**Figure 10:**
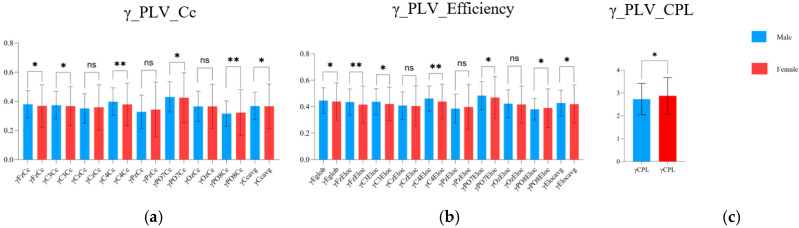
Features in the properties of the PLV network in the gamma band between the male and female groups in the aiming period. (**a**) Sex characteristics in the gamma PLV Cc; (**b**) Sex characteristics in the gamma PLV efficiency; and (**c**) Sex characteristics in the gamma PLV CPL (ns indicates *p* > 0.05, * indicates *p* ≤ 0.05, ** indicates *p* ≤ 0.01).

As shown in Figure 10, In the gamma band, the Cc values at Fz, C3, C4, PO7, and PO8; the Eglob value; the Eloc values at Fz, C3, C4, PO7, and PO8; and the average Cc and Eloc values were significantly higher in the males than in the females (*p* ≤ 0.0479). Moreover, the CPL in the gamma band was significantly lower in the male group than in the female group (*p* ≤ 0.0495), (Table 14 and Table 15).

We also calculated the PSD and the PLV of novice shooters in the theta, alpha, and beta bands during the resting state and computed the PLV network properties. The Wilcoxon rank-sum test assessed the differences in brain activity properties across different states. The results are shown in Supplementary Materials, Figures S1–S12.

### 3.5. Correlation Analysis of the Cerebral Analysis Results and Shooting Scores Between Sexes

Based on the normality test results, we used Spearman’s correlation to assess the relationship between brain neural activity and shooting score, as shown in Table 16.

We also assessed the correlation between the PLV and shooting score, showing significant results in Table 17.

### 3.6. Correlation Analysis of VAJC Results and Shooting Scores

**Figure 11:**
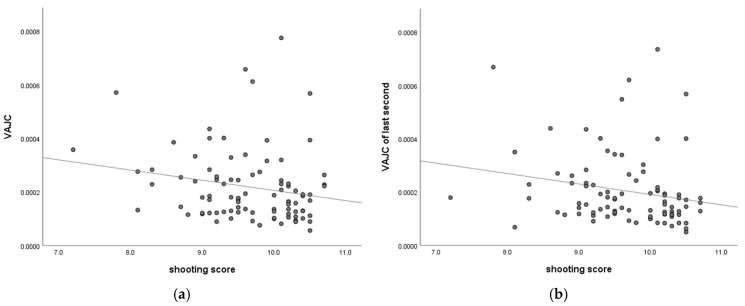
Correlation between shooting score and VAJC in the male group. (**a**) Correlation between shooting score and VAJC in males; and (**b**) Correlation between shooting score and VAJC of last second in males.

We calculated correlations between the VAJC and shooting score, showing significant results in Figure 11.

The correlation between the VAJC of the last two seconds and shooting score is shown in Figure 11a, while the correlation between the VAJC in the last second and shooting score is shown in Figure 11b.

As shown in Figure 11, the correlation between the VAJC score and shooting score in the male group was significant, whereas in the female group, the correlation was not significant (*p* > 0.05). We also computed the correlation between the shooting score and VAJC in all subjects and the female group; however, the correlation was insignificant (*p* > 0.05) (Table 18).

## 4. Discussion

### 4.1. Sex Characteristics in Aiming Stability

We analyzed the sex characteristics in the VAJC and its correlations with shooting score in the study. The VAJC value was used to assess aiming stability, with a higher VAJC value indicating poorer stability. As shown in Figure 4, VAJC values were higher in males than in females, indicating lower aiming stability in male subjects. These results align with those of Hiroshi et al., who reported significantly greater hand stability in females than males under low-force conditions [33]. Previous research has indicated that the more excellent postural stability in females can be attributed to several factors, including greater diligence and focus on postural tasks [34], lower body weight [35], anatomical features such as a lower center of gravity [36], and better trainability of the postural regulation system [37]. Jo et al. investigated sex differences in static postural control under different vision and task conditions. They reported that male’s postural stability was weaker than that of females under the same condition [38]. Our correlation analysis (see Figure 11) revealed that the VAJC was associated with shooting scores, indicating that more excellent stability (i.e., a lower VAJC) is associated with better shooting scores in male subjects. However, no significant correlation was observed between the VAJC and the shooting scores of female subjects. Previous research has shown that body sway and rifle sway reductions improve shooting accuracy and scores [39]. Shooting stability, which involves a steady posture and precise aiming, is crucial for the shooting scores [40] and can differentiate between low and high scores [41]. This finding indicated differences between males and females in the correlation between aiming stability and shooting scores. Farenc et al. investigated the influence of sex differences on an upright stance and found that females have a smaller sway amplitude for motions than males. The properties of the muscle physiology can explain this phenomenon. This finding is consistent with our experimental results, which show that females’ stability is higher than males’ [42].

### 4.2. Sex Characteristics of PSD

Changes in amplitude or power at specific electrode locations generally reflect the activation or inhibition of regional brain functions [10]. PSD EEG topographic maps in the aiming period are shown in Figure 6.

Figure 5 and Figure 6 show that the PSD results indicated different sex characteristics in the theta, alpha, alpha-2, beta, and gamma bands during the aiming process. In Figure 5, the PSD values of females were higher than those of males in the theta, alpha, and beta bands. Additionally, the PSD values of females in alpha-2 were higher than those of males at all electrodes except Oz. Haufler observed a negative correlation between theta and alpha power and brain activation, while beta and gamma power were positively correlated with activation [43]. Therefore, during the aiming process, females displayed lower brain activation in the theta, alpha, and alpha-2 bands but higher activation in the beta and gamma bands. Activity in the theta and alpha bands is a reliable indicator of adult brain arousal and attention allocation [44]. Gómez further demonstrated that attention modulation is reflected by a decrease in alpha power and an increase in beta power [45]. EEG alpha-2 power is associated with the adaptive inhibition of task-irrelevant neural processes [46,47,48,49,50], and the amplitude of alpha-2 serves as a marker of conscious self-control over cognitive and affective functions [31], suggesting that, during the aiming period, the female group exhibited stronger conscious self-control than males. Moreover, Marrufo reported that a reduction in alpha-band activity and an increase in beta-band activity are markers of visuospatial attention [51]. Beta oscillation is the carrier of visual attention [52] and is crucial in the contextual gating of visual and proprioceptive action feedback [53]. Gamma oscillations serve a variety of functions in motor control, not just signaling the execution of movements [54]. Additionally, movement-related changes in gamma oscillations from sensorimotor areas are involved in processing afferent proprioceptive feedback to control movements [55].

The results of the correlation analysis (see Table 16) revealed an associated correlation between novices’ shooting scores and the neural activity of the occipital, frontal, and central regions. Specifically, male shooting scores showed a negative association with theta band activation in the occipital lobe, a positive correlation with alpha band activation in the central area, a negative correlation with the alpha-2 PSD value, and a negative correlation with beta band activation in the central area. Moreover, a negative correlation was observed between female shooting scores and alpha band activation in both the central and occipital regions, and a positive correlation was observed with shooting scores in the alpha-2 band, indicating that higher alpha-2 PSD amplitude corresponded to better scores in females. At the same time, a positive correlation was found with beta band activation in the frontal and occipital lobes. Novices’ scores depend on the combined effects of visual function, information processing, and hand-control coordination, consistent with the findings of a previous study by Gong et al. [10]. The occipital lobe correlation across all subjects suggests that visual attention activation during the aiming process plays a crucial role in novice shooters’ scores. Increased alpha power over the occipital cortex has been associated with suppressing irrelevant visual information [56], which may explain the negative correlation between female shooting scores and alpha power in the occipital lobe. Moreover, novices manage their attentional resources through theta power in the frontal lobe during aiming [57] (see Figure S13). EEG alpha-2 amplitude is a marker of conscious self-control over cognitive and affective functions [31]. The results in the alpha-2 band may suggest that, in shooting competitions, excessive self-control in males might lead to lower scores, while females might employ moderate self-control to inhibit task-irrelevant neural processes, thereby improving their scores. Meanwhile, alpha and beta activity recorded in the central regions indicates brain processes related to hand movement [58]. The correlation between activity in the central region and performance across sexes suggests that the effective regulation of hand movements is also crucial. Previous research has also indicated that the side of the performing hand (right or left) has localized effects on recordings from the sensorimotor hand area (C3, C4) [10,59]. The stability results indicated that males exhibited low stability. Regarding the correlations between the C3 and C4 PSD and shooting score, the central region PSD reflects the influence of hand movement. Therefore, the hand-movement regulation in males may have been weaker than in females (Figure 5). The correlation between frontal lobe activity and score indicates that the brain’s information decoding and processing capabilities during the aiming process also contribute to shooting scores. Human motor cortical gamma oscillations play a key role in movement and are related to learning. The correlation results of gamma PSD in males and females indicated that the gamma PSD values are associated with shooting scores, suggesting that movement-related changes in gamma amplitude may result from motor learning in the motor cortex during movement [55].

Additionally, we compared the PSD between the task and resting states, as shown in Figures S10–S12. The results indicate that power in the theta and alpha bands is generally higher during the resting state than during the task state, except for the Oz electrode in the beta band, where the task-related PSD is higher than the resting state. The results suggest that brain activity in the theta and alpha bands is reduced during the aiming, while the beta band activity at Oz is enhanced during the task, possibly reflecting increased attention during aiming.

### 4.3. Sex Characteristics of PLV Network

PLV networks indicate the synchronization of oscillations. Studies have shown that skilled shooters with better scores present lower Cc and Eloc values, shorter CPL values, and higher Eglob values during aiming. Compared with females, males exhibited higher values of indicators such as Cc, Eglob, and Eloc but lower values of CPL. When combined with the shooting scores of different sex groups, the findings align with the correlation results of Gong et al. (to some extent) [28]. Studies have shown that the mean Cc and Eloc values reflect the efficiency of local information processing and transformation within a network, whereas the CPL and Eglob values reflect the efficiency of global information transmission within the brain network [60]. The increases in the Cc, Eglob, and Eloc values and the decrease in the CPL value indicate enhanced information processing efficiency in the brain [61]. The results (Figure 7 and Figure 8) suggest that males exhibit greater brain information processing efficiency in local and global brain regions than females during aiming.

Based on the correlation between PLV network properties and scores, we found that the active brain regions during aiming differed between male and female shooting novices. In males, the transmission efficiencies of the parietal lobe in the alpha and beta bands were negatively associated with the shooting score, while transmission efficiencies of the parietal lobe of the gamma band were positively associated with scores (see Table 17). Moreover, in females, the correlation between the transmission efficiency of the occipital lobe and the shooting score was significantly negative, and the Cc of the occipital lobe in the gamma band was positively associated with scores (see Table 17). The results of the correlation between network properties and shooting scores across different sexes may indicate that shooters with better scores tend to exhibit lower neural activity during aiming. The parietal lobe is primarily involved in sensorimotor information processing, while the occipital lobe plays a key role in visual processing and visual-spatial tasks [62,63]. Our study suggests that shooting novices of different sexes in our sample processed the same task in different brain areas. Males’ shooting scores may be associated with sensorimotor integration, primarily involving the parietal lobe, while females’ shooting scores might be associated with visual-spatial processing, engaging the occipital lobe more prominently.

Additionally, we compared the characteristics of the PLV network between the resting and the task states, as shown in Figures S1–S9. The changes in the PLV network indicate that the Cc, Eglob, and Eloc values are higher in the task state than the resting state, while the CPL value is lower in the resting state.

### 4.4. Sex Characteristics of the Shooting Score

As shown in Figure 3, the results indicate no significant sex differences in the scores of novice shooters. Previous studies have also reported no significant sex differences in shooting scores [64,65,66] Our findings are consistent with these studies, although all the shooters were novices. By combining the stability, PSD, and PLV network property analysis results, the lack of significant sex differences in shooting scores may be attributed to the following factors: (1) females exhibit better aiming stability than males in terms of behavioral performance; (2) based on the PSD results in the alpha and beta bands from the central region, females demonstrate better hand movement regulation and visual attention activation than males; and (3) males also exhibit higher information processing efficiency in both local and global brain regions compared to females. Moreover, regarding the correlation between the PLV network properties and scores, shooters with better shooting scores exhibited lower neural activity during aiming, regardless of sex, which aligns with the neural efficiency hypothesis.

### 4.5. Limitations

This study has several limitations. First, the sample size in the experiment is small. Second, since the study was conducted during a shooting competition, we employed the portable wireless EEG system to minimize potential interference in order to decrease the influence on subjects. However, compared to traditional wired systems, the portable device has a limited number of channels. Third, the elimination mechanism in the competition would remove shooters with poor scores after each round, resulting in an imbalanced sample.

## 5. Conclusions

In conclusion, as a case study, we investigated novice shooters’ neural activity and behavioral characteristics in a competition. Concerning neural activity, females exhibited greater values in PSD and CPL. In comparison, males exhibited greater values in Cc, Eloc, and Eglob, suggesting that the higher PSD values observed in female novice shooters had a specific positive effect on their scores in the shooting competition. In contrast, male novices’ higher global and local information transmission efficiency did not appear to promote their scores. In the PSD of gamma, the PSD values in the occipital and central areas of the novice shooters also affected shooting scores. In terms of aiming stability, males had lower aiming stability than females, indicating that females may have better behavioral performance than males during competition. In the future, we will strive to organize larger-scale competitions to obtain more and to conduct in-depth research.

## Figures and Tables

**Table 1:** The shooting median scores of sexes.

Sex	Median Score
Male	9.7
Female	9.9

**Table 2:** The VAJC average values of sexes.

Sex	VAJC of the Last Two Seconds	VAJC of last One Second	VAJC of Penultimate Second
Male	0.00018	0.0001640	0.0001870
Female	0.0001197	0.0001130	0.0001436

**Table 3:** The PSD average value of sexes in the theta band (dB).

Sex	θFz	θC3	θCz	θC4	θPz	θPO7	θOz	θPO8
Male	−0.3496	−2.087	−2.238	−0.3623	−6.019	−0.2448	1.309	−0.3021
Female	3.014	−1.319	−1.162	−0.5263	−3.565	1.935	2.543	1.245

**Table 4:** The PSD average value of sexes in the alpha band (dB).

Sex	αFz	αC3	αCz	αC4	αPz	αPO7	αOz	αPO8
Male	−2.585	−4.487	−5.053	−2.364	−8.611	−2.499	−0.3475	−2.470
Female	−0.1233	−3.146	−3.673	−2.604	−6.058	0.1379	0.6435	−0.9375

**Table 5:** The PSD average value of sexes in the alpha-2 band (dB).

Sex	α2Fz	α2C3	α2Cz	α2C4	α2Pz	α2PO7	α2Oz	α2PO8
Male	3.889	0.7983	−0.6692	0.8379	−2.965	4.029	5.946	3.401
Female	5.018	2.141	0.3982	1.458	−2.331	5.274	4.179	3.636

**Table 6:** The PSD average value of sexes in the beta band (dB).

Sex	βFz	βC3	βCz	βC4	βPz	βPO7	βOz	βPO8
Male	−7.102	−8.157	−10.09	−6.924	−13.71	−6.722	−4.091	−6.744
Female	−5.329	−8.422	−8.938	−6.969	−11.74	−4.550	−3.464	−5.168

**Table 7:** The PSD average value of sexes in the gamma band (dB).

Sex	γFz	γC3	γCz	γC4	γPz	γPO7	γOz	γPO8
Male	5.093	2.698	0.3612	5.247	−3.282	6.897	9.515	5.436
Female	6.847	5.235	0.4364	3.133	−3.369	5.683	6.233	5.299

**Table 8:** The Cc average values of sexes in the theta band.

Sex	Fz	C3	Cz	C4	Pz	PO7	Oz	PO8	Avg
Male	0.4907	0.4477	0.4839	0.4294	0.4022	0.4940	0.5042	0.4810	0.4700
Female	0.4595	0.4265	0.4629	0.4068	0.3998	0.4421	0.4588	0.4324	0.4400

**Table 9:** The efficiency average values of sexes in the theta band.

Sex	Eglob	Fz	C3	Cz	C4	Pz	PO7	Oz	PO8	Avg	CPL
Male	0.5204	0.5171	0.4681	0.5137	0.4599	0.4443	0.5221	0.5303	0.5076	0.4938	2.266
Female	0.4940	0.4909	0.4581	0.4917	0.4456	0.4345	0.4766	0.4890	0.4665	0.4645	2.378

**Table 10:** The Cc average values of sexes in the alpha band.

Sex	Fz	C3	Cz	C4	Pz	PO7	Oz	PO8	Avg
Male	0.4554	0.3845	0.4357	0.3817	0.3415	0.4493	0.4672	0.4332	0.4152
Female	0.3976	0.3536	0.4096	0.3596	0.3300	0.3803	0.3977	0.3523	0.3692

**Table 11:** The efficiency average values of sexes in the alpha band.

Sex	Eglob	Fz	C3	Cz	C4	Pz	PO7	Oz	PO8	Avg	CPL
Male	0.4375	0.4752	0.4181	0.4680	0.4212	0.3856	0.4764	0.4892	0.4602	0.4505	2.509
Female	0.4298	0.4351	0.3861	0.4397	0.3914	0.3639	0.4115	0.4262	0.3889	0.4078	2.756

**Table 12:** The Cc average values of sexes in the beta band.

Sex	Fz	C3	Cz	C4	Pz	PO7	Oz	PO8	Avg
Male	0.4320	0.3707	0.4112	0.3639	0.2802	0.4101	0.4386	0.3882	0.3820
Female	0.3432	0.2925	0.3549	0.3008	0.2470	0.3013	0.3305	0.2856	0.3097

**Table 13:** The efficiency average values of sexes in the beta band.

Sex	Eglob	Fz	C3	Cz	C4	Pz	PO7	Oz	PO8	Avg	CPL
Male	0.4402	0.4607	0.4014	0.4477	0.3878	0.3306	0.4364	0.4662	0.4247	0.4173	2.742
Female	0.3647	0.3763	0.3370	0.3857	0.3437	0.2953	0.3388	0.3671	0.3336	0.3457	3.267

**Table 14:** The Cc average values of sexes in the gamma band.

Sex	Fz	C3	Cz	C4	Pz	PO7	Oz	PO8	Avg
Male	0.3722	0.3666	0.3519	0.4099	0.3002	0.4346	0.3547	0.2987	0.3607
Female	0.3288	0.3144	0.2987	0.3251	0.2553	0.3624	0.3129	0.2573	0.3020

**Table 15:** The Efficiency average values of sexes in the gamma band.

Sex	Eglob	Fz	C3	Cz	C4	Pz	PO7	Oz	PO8	Avg	CPL
Male	0.4500	0.4350	0.4400	0.4150	0.4700	0.3700	0.4900	0.4200	0.3600	0.4300	2.622
Female	0.3855	0.3665	0.3791	0.3532	0.3888	0.3278	0.4094	0.3678	0.3340	0.3671	2.983

**Table 16:** The significant correlations between the shooting score and PSD according to sex in the case.

	PSD		
		R	*p*
Male	thetaPO8	0.223	0.035
	alphaC3	−0.273	0.010
	alphaC4	−0.220	0.039
	alpha-2PO7	−0.278	0.0087
	betaC3	−0.261	0.014
	betaC4	−0.249	0.019
	gammaC3	−0.230	0.031
	gammaC4	−0.320	0.002
Female	alphaC4	0.242	0.005
	alphaPO7	0.266	0.002
	alpha-2PO7	0.188	0.028
	betaFz	0.171	0.046
	betaPO7	0.249	0.003
	gammaC3	0.208	0.015
	gammaPO8	0.233	0.006

**Table 17:** The significant correlations between the shooting score and PLV according to sex in the case.

	PLV		
		R	*p*
Male	alphaPz_Cc	−0.254	0.016
	alphaPz_Eloc	−0.267	0.011
	betaPz_Cc	−0.328	0.002
	betaPz_Eloc	−0.352	0.001
	gammaC4Eloc	0.216	0.043
	gammaPzEloc	0.224	0.036
Female	alphaPO8_Eloc	−0.178	0.038
	betaOz_Cc	−0.169	0.050
	betaOz_Eloc	−0.22	0.010
	gammaPO8Cc	0.191	0.026

**Table 18:** Correlations between shooting score and VAJC according to sex in the study.

		R	*p*
Male	VAJC	−0.234	0.026
	VAJC of last second	−0.281	0.007
Female	VAJC	−0.073	0.39
	VAJC of last second	−0.103	0.228

## Data Availability

The raw data supporting the conclusions of this article will be made available by the authors, without undue reservation.

## Supplementary Materials

The following supporting information can be downloaded at: https://www.mdpi.com/article/10.3390/brainsci15020174/s1, Figure S1: Differences of clustering coefficient (Cc) values of brain network of theta band between rest and task; Figure S2: Differences of efficiency (Eglob, Eloc) values of brain network of theta band between rest and task; Figure S3: Differences of characteristic path length (CPL) values of brain network of theta band between rest and task; Figure S4: Differences of Cc values of brain network of alpha band between rest and task; Figure S5: Differences of Eglob and Eloc values of brain network of alpha band between rest and task; Figure S6: differences of CPL values of brain network of alpha band between rest and task; Figure S7: Differences of Cc values of brain network of beta band between rest and task; Figure S8: Differences of Eglob and Eloc values of brain network of beta band between rest and task; Figure S9: Difference of CPL values of brain network of beta band between rest and task; Figure S10: Difference of PSD values of theta band between rest and task; Figure S11: Difference of PSD values of alpha band between rest and task; Figure S12: Difference of PSD values of beta band between rest and task; Figure S13: The correlation between shooting score and PSD values in all players. (a) shows a significantly positive correlation between the Fz PSD value in the theta band and the shooting scores (rs = 0.142, *p* < 0.05). (b) shows a significant positive correlation between the PO7 PSD value in the theta band and the shooting scores (rs = 0.160, *p* < 0.05); (c) shows a significantly positive correlation between the PO7 PSD value and shooting score in the alpha band (rs = 0.182, *p* < 0.01), (d) shows in the beta band of all participants, a significant positive correlation between the PSD value at PO7 electrode and scores (rs= 0.179, *p* < 0.01); Figure S14: The correlation between shooting score and PLV network properties values in all subjects. (a) shows a significantly negative correlation between alpha PO8 Cc values and shooting scores (rs = −0.132, *p* < 0.05); (b) shows a significantly negative correlation between alpha PO8 Eloc values and shooting scores (rs = −0.147, *p* < 0.05); (c) shows a significantly negative correlation between beta Pz Eloc values and shooting scores (rs = −0.132, *p* < 0.05); (d) shows a significantly negative correlation between beta Oz Eloc values and shooting scores (rs = −0.134, *p* < 0.05); Table S1: The results of normality test of aiming stability data; Table S2: The results of normality test of PSD values; Table S3: The results of normality test of PLV values.
