# Supplementary material for: A Case Study on Neural Activity Characteristics in a Shooting Competition
Source: Brain Sci. 2025 Feb 10;15(2):174. doi: 10.3390/brainsci15020174 (PMC11853501; doi:10.3390/brainsci15020174)
Supplement: Supplementary file 1 [file brainsci-15-00174-s001.zip › brainsci-3421822-supplementary.pdf]

Supplementary Materials

Figure S1-S12 are gender differences of the PLV network properties values and PSD values in resting state. **Figure S13-S14** are correlations between shooting scores and PLV and PSD values in all players. **Table S1-S3** are results of normality test of behavioral, brain properties datasets.

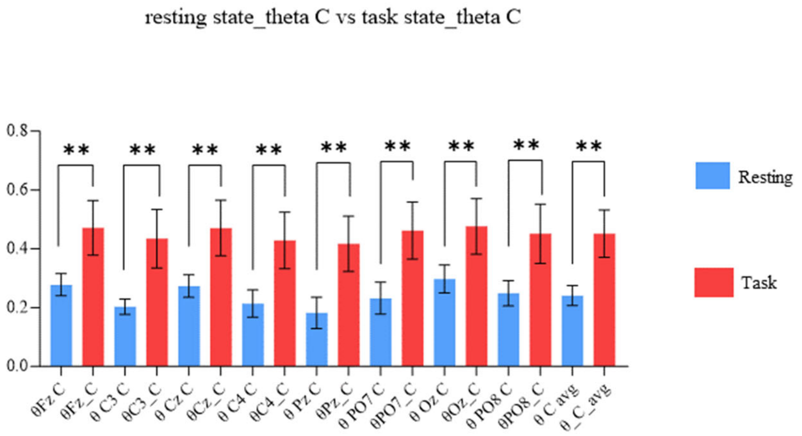

Figure S1. Differences of clustering coefficient (Cc) values of brain network of theta band between rest and task. From the **Figure S1**, the task's brain network properties were greater than rest's in the theta band.

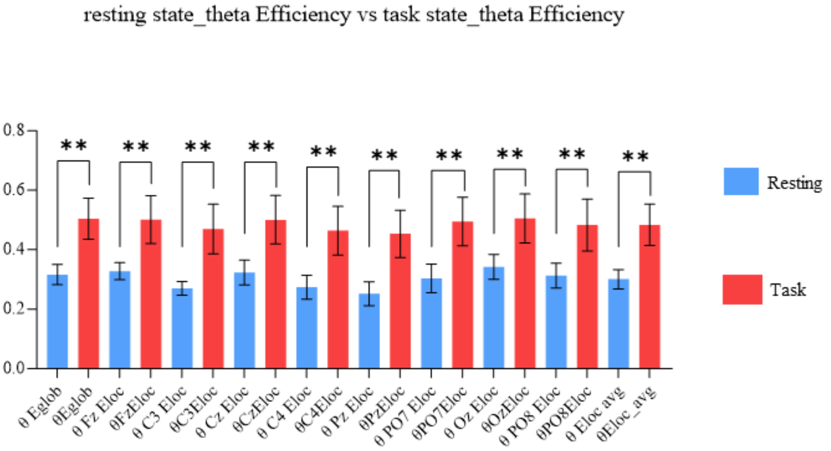

Figure S2. Differences of efficiency (Eglob, Eloc) values of brain network of theta band between rest and task. From the **Figure S2**, the task's brain network properties were greater than rest's in the theta band.

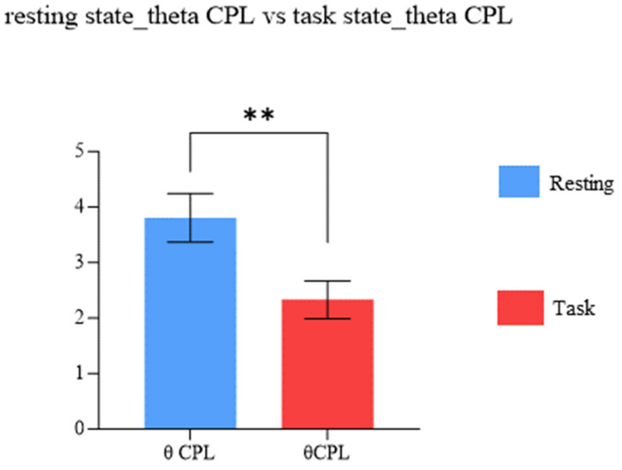

Figure S3. differences of characteristic path length (CPL) values of brain network of theta band between rest and task. From the **Figure S3**, the task's brain network properties were lower than rest's in the theta band.

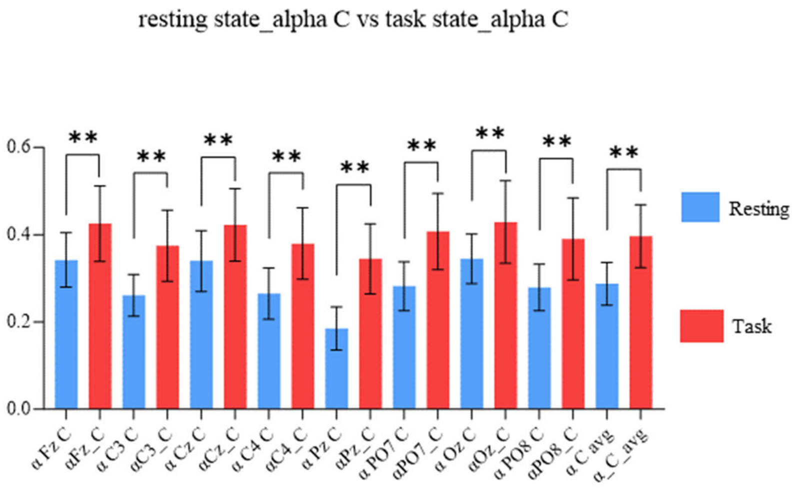

Figure S4. Differences of Cc values of brain network of alpha band between rest and task. From the **Figure S4**, the task's brain network properties were greater than rest's in the alpha band.

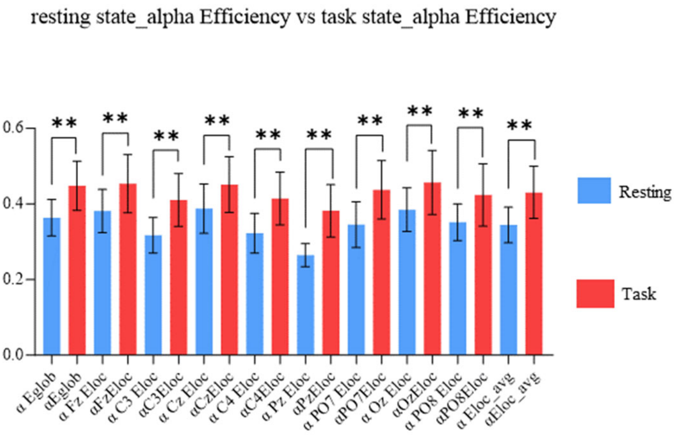

Figure S5. Differences of Eglob and Eloc values of brain network of alpha band between rest and task. From the **Figure S5**, the task's brain network properties were greater than rest's in the theta band.

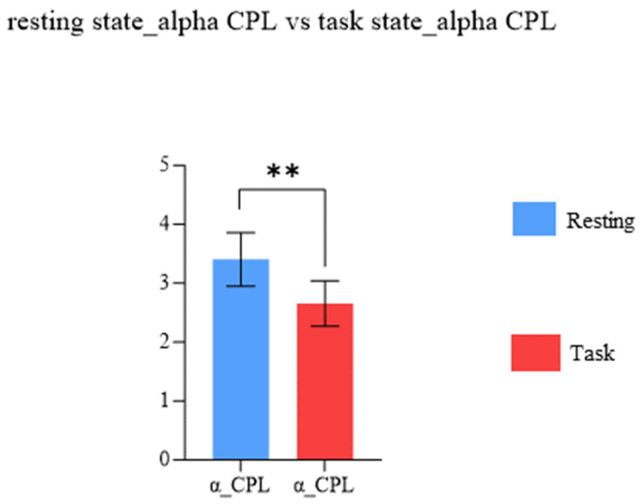

Figure S6. differences of CPL values of brain network of alpha band between rest and task. From the **Figure S6**, the task's brain network properties were lower than rest's in the alpha band.

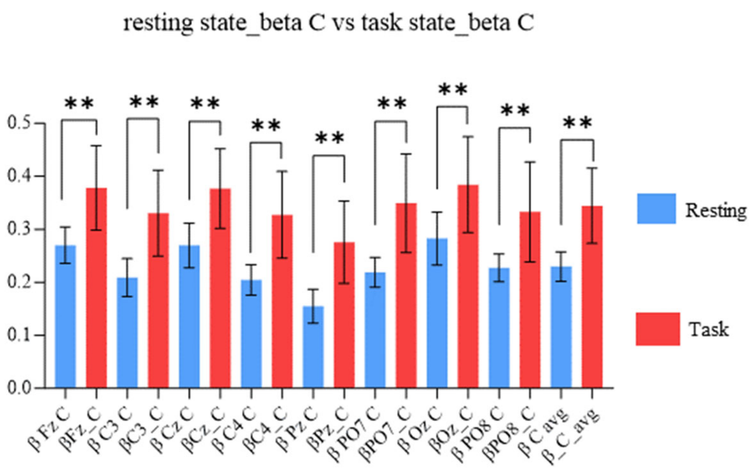

Figure S7. Differences of Cc values of brain network of beta band between rest and task. From the **Figure S7**, the task's brain network properties were greater than rest's in the beta band.

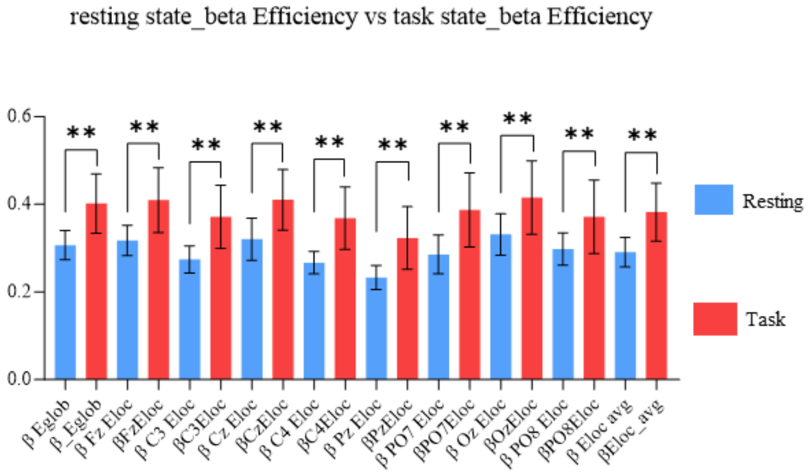

Figure S8. Differences of Eglob and Eloc values of brain network of beta band between rest and task. From the **Figure S8**, the task's brain network properties were greater than rest's in the beta band.

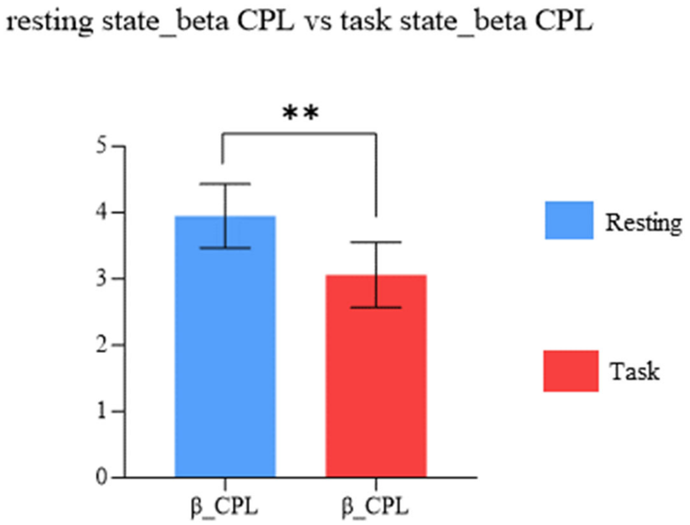

Figure S9. Difference of CPL values of brain network of beta band between rest and task. From the **Figure S9**, the task's brain network properties were lower than rest's in the beta band.

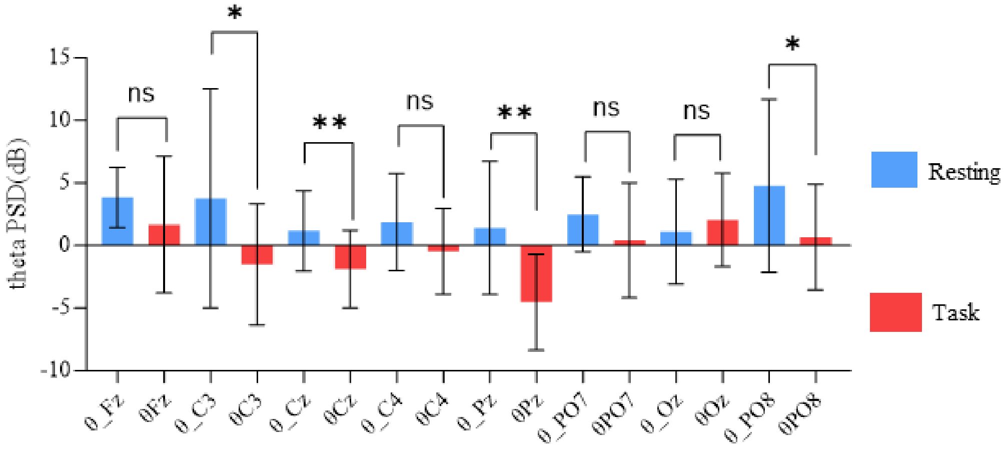

Figure S10. Difference of PSD values of theta band between rest and task. From the **Figure S10**, the task's PSD values were lower than rest's in the theta band.

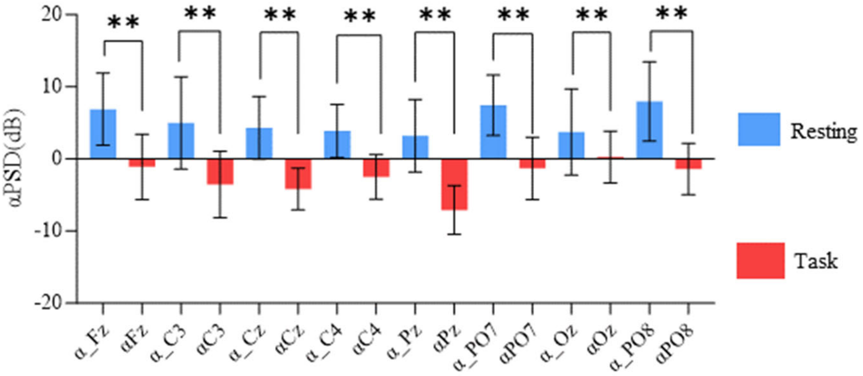

Figure S11. Difference of PSD values of alpha band between rest and task. From the **Figure S11**, the task's PSD values were lower than rest's in the alpha band.

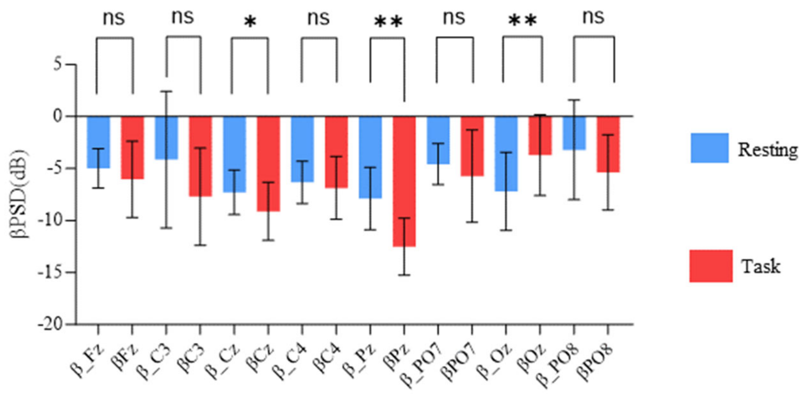

Figure S12. Difference of PSD values of beta band between rest and task. From the **Figure S12**, the task's PSD values were lower than rest's in the beta band, and the PSD value at Oz in task was greater than rest's in the beta band.

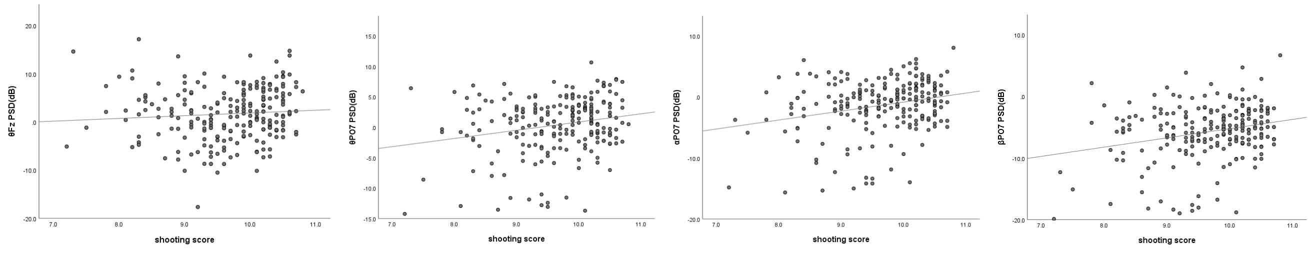

(a) **(b)** **(c)** **(d)**

Figure S13. The correlation between shooting score and PSD values in all players. (**a**) shows a significantly positive correlation between the Fz PSD value in the theta band and the shooting scores (r_s= 0.142, p<0.05). (**b**) shows a significant positive correlation between the PO7 PSD value in the theta band and the shooting scores (r_s= 0.160, p<0.05); (**c**) shows a significantly positive

correlation between the PO7 PSD value and shooting score in the alpha band (r_s= 0.182, $p<0.01$), (**d**) shows in the beta band of all participants, a significant positive correlation between the PSD value at PO7 electrode and scores (r_s= 0.179, $p<0.01$).

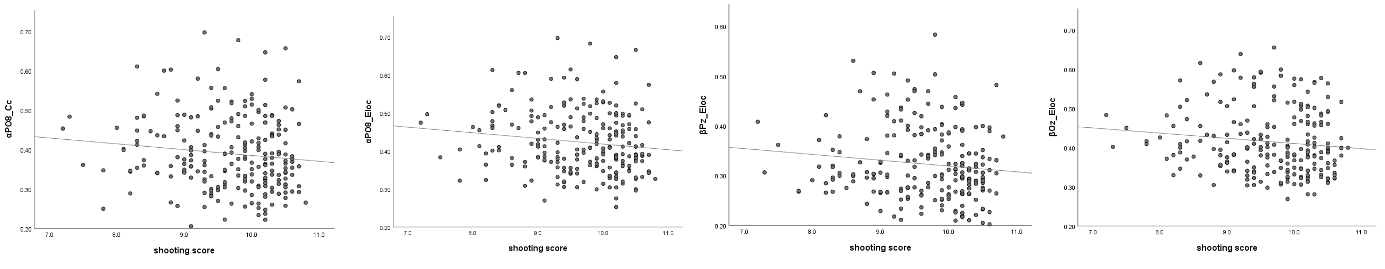

(**a**) (**b**) (**c**) (**d**)

Figure S14. The correlation between shooting score and PLV network properties values in all subjects. (**a**) shows a significantly negative correlation between alpha PO8 Cc values and shooting scores (r_s= -0.132, $p<0.05$); (**b**) shows a significantly negative correlation between alpha PO8 Eloc values and shooting scores (r_s= -0.147, $p<0.05$); (**c**) shows a significantly negative correlation between beta Pz Eloc values and shooting scores (r_s= -0.132, $p<0.05$); (**d**) shows a significantly negative correlation between beta Oz Eloc values and shooting scores (r_s= -0.134, $p<0.05$).

Table S1. The results of normality test of aiming stability data.

dataset	Shapiro-Wilk p value	Kolmogorov-Smirnov p value	Normality test
Score(M)	0.0003	0.0013	Non-normal distribution
Score(F)	<0.0001	<0.0001	Non-normal distribution
VACJ of last two seconds(M)	<0.0001	<0.0001	Non-normal distribution
VACJ of last two seconds(F)	<0.0001	<0.0001	Non-normal distribution
VACJ of last one second(M)	<0.0001	<0.0001	Non-normal distribution
VACJ of last one second(F)	<0.0001	<0.0001	Non-normal distribution
VACJ of penultimate second(M)	<0.0001	0.0006	Non-normal distribution
VACJ of penultimate second(F)	<0.0001	<0.0001	Non-normal distribution

Table S2. The results of normality test of PSD values.

Dataset(PSD)	Shapiro-Wilk p value	Kolmogorov-Smirnov p value	Normality test
thetaFz(M)	0.1337	> 0.1	Normal distribution
thetaFz(F)	0.7639	> 0.1	Normal distribution
thetaC3(M)	0.0289	0.0854	Non-normal distribution
thetaC3(F)	< 0.0001	< 0.0001	Non-normal distribution
thetaCz(M)	0.8	> 0.1	Normal distribution
thetaCz(F)	0.0078	> 0.1	Non-normal distribution
thetaC4(M)	0.4708	> 0.1	Normal distribution
thetaC4(F)	0.8363	> 0.1	Normal distribution
thetaPz(M)	0.5819	> 0.1	Normal distribution
thetaPz(F)	0.7612	> 0.1	Normal distribution
thetaPO7(M)	0.0003	0.0176	Non-normal distribution
thetaPO7(F)	0.0076	> 0.1	Non-normal distribution
thetaOz(M)	0.5180	> 0.1	Normal distribution
thetaOz(F)	0.8836	> 0.1	Normal distribution
thetaPO8(M)	0.2228	> 0.1	Normal distribution
thetaPO8(F)	0.0016	0.0154	Non-normal distribution
alphaFz(M)	0.0841	> 0.1	Normal distribution
alphaFz(F)	0.2428	> 0.1	Normal distribution

alphaC3(M)	0.5808	> 0.1	Normal distribution
alphaC3(F)	< 0.0001	< 0.0001	Non-normal distribution
alphaCz(M)	0.0486	> 0.1	Non-normal distribution
alphaCz(F)	< 0.0001	> 0.1	Non-normal distribution
alphaC4(M)	0.2129	> 0.1	Normal distribution
alphaC4(F)	0.5713	> 0,1	Normal distribution
alphaPz(M)	0.7557	> 0.1	Normal distribution
alphaPz(F)	0.8484	> 0.1	Normal distribution
alphaPO7(M)	< 0.0001	0.0002	Non-normal distribution
alphaPO7(F)	0.1235	> 0.1	Normal distribution
alphaOz(M)	0.3955	0.0682	Normal distribution
alphaOz(F)	0.7666	> 0.1	Normal distribution
alphaPO8(M)	0.3123	> 0.1	Normal distribution
alphaPO8(F)	0.0008	0.004	Non-normal distribution
betaFz(M)	0.1455	> 0.1	Normal distribution
betaFz(F)	0.3197	> 0.1	Normal distribution
betaC3(M)	0.3913	> 0.1	Normal distribution
betaC3(F)	< 0.0001	< 0.0001	Non-normal distribution
betaCz(M)	< 0.0001	< 0.0001	Non-normal distribution
betaCz(F)	0.0153	> 0.1	Normal distribution
betaC4(M)	0.0037	> 0.1	Non-normal distribution
betaC4(F)	0.0884	> 0.1	Normal distribution
betaPz(M)	0.2197	> 0.1	Normal distribution
betaPz(F)	0.0968	> 0.1	Normal distribution
betaPO7(M)	0.0002	< 0.0001	Non-normal distribution
betaPO7(F)	0.0093	0.0222	Non-normal distribution
betaOz(M)	0.8900	> 0.1	Normal distribution
betaOz(F)	0.1232	> 0.1	Normal distribution
betaPO8(M)	0.3171	> 0.1	Normal distribution
betaPO8(F)	< 0.0001	0.0017	Non-normal distribution
gammaFz(M)	0.1431	> 0.1	Normal distribution
gammaFz(F)	< 0.0001	<0.0001	Non-normal distribution
gammaC3(M)	0.0061	0.0773	Non-normal distribution
gammaC3(F)	< 0.0001	< 0.0001	Non-normal distribution
gammaCz(M)	0.0005	0.0016	Non-normal distribution
gammaCz(F)	<0.0001	<0.0001	Non-normal distribution
gammaC4(M)	0.0342	0.0436	Non-normal distribution
gammaC4(F)	0.0081	0.0352	Non-normal distribution
gammaPz(M)	0.0043	> 0.1	Non-normal distribution
gammaPz(F)	< 0.0001	< 0.0001	Non-normal distribution
gammaPO7(M)	0.0173	0.0304	Non-normal distribution
gammaPO7(F)	0.0517	0.0038	Non-normal distribution
gammaOz(M)	0.4275	> 0.1	Normal distribution
gammaOz(F)	0.0438	> 0.1	Non-normal distribution
gammaPO8(M)	0.0005	> 0.1	Non-normal distribution
gammaPO8(F)	0.0014	< 0.0001	Non-normal distribution

Table S3. The results of normality test of PLV values.

Dataset(PLV)	Shapiro-Wilk p value	Kolmogorov-Smirnov p value	Normality test
thetaFz_Cc(M)	0.6891	> 0.1	Normal distribution
thetaFz_Cc(F)	0.3997	> 0.1	Normal distribution
thetaC3_Cc(M)	0.3894	> 0.1	Normal distribution
thetaC3_Cc(F)	0.0853	> 0.1	Normal distribution
thetaCz_Cc(M)	0.8138	> 0.1	Normal distribution
thetaCz_Cc(F)	0.9048	> 0.1	Normal distribution
thetaC4_Cc(M)	0.0176	> 0.1	Non-normal distribution
thetaC4_Cc(F)	0.0148	0.0066	Non-normal distribution
thetaPz_Cc(M)	0.0083	0.0063	Non-normal distribution
thetaPz_Cc(F)	0.0106	0.0653	Non-normal distribution
thetaPO7_Cc(M)	0.6244	> 0.1	Normal distribution
thetaPO7_Cc(F)	0.2772	> 0.1	Normal distribution
thetaOz_Cc(M)	0.2588	> 0.1	Normal distribution
thetaOz_Cc(F)	0.4166	> 0.1	Normal distribution
thetaPO8_Cc(M)	0.9824	> 0.1	Normal distribution
thetaPO8_Cc(F)	0.3613	> 0.1	Normal distribution
thetaCc_avg(M)	0.3042	> 0.1	Normal distribution
thetaCc_avg(F)	0.0125	0.0352	Non-normal distribution
thetaEglob(M)	0.6064	> 0.1	Normal distribution
thetaEglob(F)	0.1149	> 0.1	Normal distribution
thetaFz_Eloc(M)	0.4622	> 0.1	Normal distribution
thetaFz_Eloc(F)	0.5326	> 0.1	Normal distribution
thetaC3_Eloc(M)	0.0684	0.0481	Non-normal distribution
thetaC3_Eloc(F)	0.0395	> 0.1	Non-normal distribution
thetaCz_Eloc(M)	0.6715	> 0.1	Normal distribution
thetaCz_Eloc(F)	0.6429	> 0.1	Normal distribution
thetaC4_Eloc(M)	0.0042	0.0586	Non-normal distribution
thetaC4_Eloc(F)	0.0758	0.0948	Non-normal distribution
thetaPz_Eloc(M)	0.0059	0.0028	Non-normal distribution
thetaPz_Eloc(F)	0.0004	0.0038	Non-normal distribution
thetaPO7_Eloc(M)	0.2490	> 0.1	Normal distribution
thetaPO7_Eloc(F)	0.2421	> 0.1	Normal distribution
thetaOz_Eloc(M)	0.4516	> 0.1	Normal distribution
thetaOz_Eloc(F)	0.1103	> 0.1	Normal distribution
thetaPO8_Eloc(M)	0.8676	> 0.1	Normal distribution
thetaPO8_Eloc(F)	0.7265	> 0.1	Normal distribution
thetaEloc_avg(M)	0.4186	> 0.1	Normal distribution
thetaEloc_avg(F)	0.0484	0.0410	Non-normal distribution
thetaCPL(M)	0.4657	> 0.1	Normal distribution
thetaCPL(F)	0.7561	> 0.1	Normal distribution
alphaFz_Cc(M)	0.5535	> 0.1	Normal distribution
alphaFz_Cc(F)	0.0031	0.0176	Non-normal distribution
alphaC3_Cc(M)	0.0267	0.0872	Normal distribution
alphaC3_Cc(F)	0.0002	0.0216	Non-normal distribution
alphaCz_Cc(M)	0.6107	> 0.1	Normal distribution
alphaCz_Cc(F)	0.0223	> 0.1	Non-normal distribution
alphaC4_Cc(M)	0.0153	0.0139	Non-normal distribution
alphaC4_Cc(F)	0.0218	> 0.1	Non-normal distribution
alphaPz_Cc(M)	0.0888	> 0.1	Normal distribution
alphaPz_Cc(F)	0.0051	0.0674	Non-normal distribution
alphaPO7_Cc(M)	0.3762	> 0.1	Normal distribution
alphaPO7_Cc(F)	0.2949	> 0.1	Normal distribution

alphaOz_Cc(M)	0.8643	> 0.1	Normal distribution
alphaOz_Cc(F)	0.0004	0.0226	Non-normal distribution
alphaPO8_Cc(M)	0.8822	> 0.1	Normal distribution
alphaPO8_Cc(F)	< 0.0001	< 0.0001	Non-normal distribution
alphaCc_avg(M)	0.1044	0.0255	Non-normal distribution
alphaCc_avg(F)	< 0.0001	0.0035	Non-normal distribution
alphaEglob(M)	0.1339	> 0.1	Normal distribution
alphaEglob(F)	0.0002	0.0890	Non-normal distribution
alphaFz_Eloc(M)	0.2862	> 0.1	Normal distribution
alphaFz_Eloc(F)	0.0037	0.0095	Non-normal distribution
alphaC3_Eloc(M)	0.0125	> 0.1	Non-normal distribution
alphaC3_Eloc(F)	< 0.0001	0.0024	Non-normal distribution
alphaCz_Eloc(M)	0.4265	> 0.1	Normal distribution
alphaCz_Eloc(F)	0.0928	> 0.1	Normal distribution
alphaC4_Eloc(M)	0.0041	0.0139	Non-normal distribution
alphaC4_Eloc(F)	0.0010	> 0.1	Non-normal distribution
alphaPz_Eloc(M)	0.0133	0.0066	Non-normal distribution
alphaPz_Eloc(F)	0.0003	0.0574	Non-normal distribution
alphaPO7_Eloc(M)	0.2533	> 0.1	Normal distribution
alphaPO7_Eloc(F)	0.0738	> 0.1	Normal distribution
alphaOz_Eloc(M)	0.8771	> 0.1	Normal distribution
alphaOz_Eloc(F)	0.0002	0.0037	Non-normal distribution
alphaPO8_Eloc(M)	0.3565	0.0702	Normal distribution
alphaPO8_Eloc(F)	< 0.0001	< 0.0001	Non-normal distribution
alphaEloc_avg(M)	0.1159	> 0.1	Normal distribution
alphaEloc_avg(F)	< 0.0001	0.0247	Non-normal distribution
alphaCPL(M)	0.3868	> 0.1	Normal distribution
alphaCPL(F)	0.7092	> 0.1	Normal distribution
betaFz_Cc(M)	0.2114	> 0.1	Normal distribution
betaFz_Cc(F)	0.1350	> 0.1	Normal distribution
betaC3_Cc(M)	0.3036	> 0.1	Normal distribution
betaC3_Cc(F)	0.0022	> 0.1	Non-normal distribution
betaCz_Cc(M)	0.1285	> 0.1	Normal distribution
betaCz_Cc(F)	0.1212	> 0.1	Normal distribution
betaC4_Cc(M)	0.0115	0.0002	Non-normal distribution
betaC4_Cc(F)	0.2137	> 0.1	Normal distribution
betaPz_Cc(M)	0.0152	0.0392	Non-normal distribution
betaPz_Cc(F)	< 0.0001	0.0004	Non-normal distribution
betaPO7_Cc(M)	0.0865	0.0041	Non-normal distribution
betaPO7_Cc(F)	0.0720	0.0239	Non-normal distribution
betaOz_Cc(M)	0.3514	> 0.1	Normal distribution
betaOz_Cc(F)	< 0.0001	0.0165	Non-normal distribution
betaPO8_Cc(M)	0.1676	> 0.1	Normal distribution
betaPO8_Cc(F)	0.0004	0.0481	Non-normal distribution
betaCc_avg(M)	0.0109	0.0239	Non-normal distribution
betaCc_avg(F)	0.0015	> 0.1	Non-normal distribution
betaEglob(M)	0.0554	0.0614	Normal distribution
betaEglob(F)	0.0058	0.0669	Non-normal distribution
betaFz_Eloc(M)	0.1619	0.0882	Normal distribution
betaFz_Eloc(F)	0.1413	> 0.1	Normal distribution
betaC3_Eloc(M)	0.1598	0.0071	Non-normal distribution
betaC3_Eloc(F)	0.0076	> 0.1	Non-normal distribution
betaCz_Eloc(M)	0.2447	> 0.1	Normal distribution
betaCz_Eloc(F)	0.0578	> 0.1	Normal distribution
betaC4_Eloc(M)	0.0760	0.0162	Non-normal distribution

betaC4_Eloc(F)	0.0825	> 0.1	Normal distribution
betaPz_Eloc(M)	0.0068	0.0023	Non-normal distribution
betaPz_Eloc(F)	< 0.0001	< 0.0001	Non-normal distribution
betaPO7_Eloc(M)	0.0850	0.0769	Normal distribution
betaPO7_Eloc(F)	0.0076	0.0256	Non-normal distribution
betaOz_Eloc(M)	0.0878	0.0808	Normal distribution
betaOz_Eloc(F)	< 0.0001	0.0114	Non-normal distribution
betaPO8_Eloc(M)	0.2933	> 0.1	Normal distribution
betaPO8_Eloc(F)	0.0005	0.0277	Non-normal distribution
betaEloc_avg(M)	0.0377	> 0.1	Non-normal distribution
betaEloc_avg(F)	0.0046	> 0.1	Non-normal distribution
betaCPL(M)	0.3114	> 0.1	Normal distribution
betaCPL(F)	0.2467	> 0.1	Normal distribution
gammaFz_Cc(M)	0.0158	> 0.1	Non-normal distribution
gammaFz_Cc(F)	<0.0001	< 0.0001	Non-normal distribution
gammaC3_Cc(M)	0.2166	> 0.1	Normal distribution
gammaC3_Cc(F)	< 0.0001	< 0.0001	Non-normal distribution
gammaCz_Cc(M)	0.1537	> 0.1	Normal distribution
gammaCz_Cc(F)	< 0.0001	< 0.0001	Non-normal distribution
gammaC4_Cc(M)	0.0275	> 0.1	Non-normal distribution
gammaC4_Cc(F)	< 0.0001	< 0.0001	Non-normal distribution
gammaPz_Cc(M)	< 0.0001	< 0.0001	Non-normal distribution
gammaPz_Cc(F)	< 0.0001	< 0.0001	Non-normal distribution
gammaPO7_Cc(M)	0.0662	0.0961	Normal distribution
gammaPO7_Cc(F)	< 0.0001	< 0.0001	Non-normal distribution
gammaOz_Cc(M)	0.0628	> 0.1	Normal distribution
gammaOz_Cc(F)	< 0.0001	<0.0001	Non-normal distribution
gammaPO8_Cc(M)	< 0.0001	< 0.0001	Non-normal distribution
gammaPO8_Cc(F)	< 0.0001	< 0.0001	Non-normal distribution
gammaCc_avg(M)	0.0061	> 0.1	Non-normal distribution
gammaCc_avg(F)	< 0.0001	< 0.0001	Non-normal distribution
gammaEglob(M)	0.1100	> 0.1	Normal distribution
gammaEglob(F)	< 0.0001	< 0.0001	Non-normal distribution
gammaFz_Eloc(M)	0.0051	0.0002	Non-normal distribution
gammaFz_Eloc(F)	< 0.0001	< 0.0001	Non-normal distribution
gammaC3_Eloc(M)	0.1969	> 0.1	Normal distribution
gammaC3_Eloc(F)	< 0.0001	< 0.0001	Non-normal distribution
gammaCz_Eloc(M)	0.1607	> 0.1	Normal distribution
gammaCz_Eloc(F)	< 0.0001	< 0.0001	Non-normal distribution
gammaC4_Eloc(M)	0.037	> 0.1	Non-normal distribution
gammaC4_Eloc(F)	< 0.0001	< 0.0001	Non-normal distribution
gammaPz_Eloc(M)	0.029	0.0392	Non-normal distribution
gammaPz_Eloc(F)	< 0.0001	< 0.0001	Non-normal distribution
gammaPO7_Eloc(M)	0.0737	0.0903	Non-normal distribution
gammaPO7_Eloc(F)	< 0.0001	< 0.0001	Non-normal distribution
gammaOz_Eloc(M)	0.7229	> 0.1	Normal distribution
gammaOz_Eloc(F)	< 0.0001	< 0.0001	Non-normal distribution
gammaPO8_Eloc(M)	< 0.0001	0.0004	Non-normal distribution
gammaPO8_Eloc(F)	< 0.0001	< 0.0001	Non-normal distribution
gammaEloc_avg(M)	0.0889	0.0936	Normal distribution
gammaEloc_avg(F)	< 0.0001	< 0.0001	Non-normal distribution
gammaCPL(M)	0.0398	> 0.1	> 0.1
gammaCPL(F)	< 0.0001	< 0.0001	Non-normal distribution
